# DNA Damage Due to Oxidative Stress in Chronic Obstructive Pulmonary Disease (COPD)

**DOI:** 10.3390/ijms131216853

**Published:** 2012-12-10

**Authors:** Eirini Neofytou, Eleni G. Tzortzaki, Argiro Chatziantoniou, Nikolaos M. Siafakas

**Affiliations:** 1 Laboratory of Molecular and Cellular Pulmonology, Medical School, University of Crete, Crete 71110, Greece; E-Mails: niniof@gmail.com (E.N.); molecular.pneumology.lab@gmail.com (A.C.); siafak@med.uoc.gr (N.M.S.); 2 Department of Thoracic Medicine, University Hospital of Heraklion, Crete 71110, Greece

**Keywords:** chronic bronchitis, emphysema, oxidative stress, DNA damage, Microsatellite DNA, 8-OHdG, DNA repair

## Abstract

According to the American Thorasic Society (ATS)/European Respiratory Society (ERS) Statement, chronic obstructive pulmonary disease (COPD) is defined as a preventable and treatable disease with a strong genetic component, characterized by airflow limitation that is not fully reversible, but is usually progressive and associated with an enhanced inflammatory response of the lung to noxious particles or gases. The main features of COPD are chronic inflammation of the airways and progressive destruction of lung parenchyma and alveolar structure. The pathogenesis of COPD is complex due to the interactions of several mechanisms, such as inflammation, proteolytic/antiproteolytic imbalance, oxidative stress, DNA damage, apoptosis, enhanced senescence of the structural cells and defective repair processes. This review focuses on the effects of oxidative DNA damage and the consequent immune responses in COPD. In susceptible individuals, cigarette smoke injures the airway epithelium generating the release of endogenous intracellular molecules or danger-associated molecular patterns from stressed or dying cells. These signals are captured by antigen presenting cells and are transferred to the lymphoid tissue, generating an adaptive immune response and enhancing chronic inflammation.

## 1. Oxidative Stress in Chronic Obstructive Pulmonary Disease (COPD)

The respiratory tract is in direct contact with the external environment. Considering its large surface area the lung is highly prone to injury since it is exposed to high concentrations of oxygen, pollutants, toxicants and cigarette smoke [1–3]. Host factors including genetic susceptibility, epigenetic changes, and oxidative stress contribute to COPD pathogenesis by amplifying inflammation induced by inhaled toxic substances and mainly cigarette smoke [4–8].

Cigarette smoke releases thousands of oxidants, free radicals and chemical compounds, including hydroxyl radicals (−OH) and hydrogen peroxide (H_2_O_2_). Moreover, it recruits immune and inflammatory cells (e.g., alveolar macrophages, epithelial and endothelial cells, neutrophils, eosinophils, monocytes and lymphocytes) to the lungs aggravating the oxidant/antioxidant imbalance [2,3,9].

Oxidative inactivation of antiproteases and surfactants, mucus hypersecretion, membrane lipid peroxidation, mitochondrial dysfunction, alveolar epithelial injury, remodeling of extracellular matrix, altered apoptosis and cellular DNA damage are considered effects of oxidative stress in the lung [1,10]. In addition, the kinase signaling pathways induced by oxidative stress, lead to chromatin modifications (histone methylation/demethylation and histone acetylation/deacetylation) in inflammation, senescence, and steroid resistance.

Although oxidative stress, under normal circumstances, is combated by multiple antioxidant and repair systems used for the replacement of damaged cells, nucleic acids, proteins and lipids, an imbalance between oxidants and antioxidants is present in the lungs of patients with COPD [1,9,11].

## 2. Oxidative DNA Damage

Oxidative stress on cells most frequently targets the DNA. Evidence show that the continuous oxidative damage of the DNA is involved in the pathophysiology of various diseases and conditions such as cancer, atherosclerosis, neurodegenerative disorders, chronic lung diseases, aging [12].

The distribution of oxidative damage in the genome depends on the varying susceptibility of sequences to oxidative attack, and the preferential targeting of repair processes. Non-coding DNA (microsatellite sequences-MS, telomeres, promoters and sites of methylation), is particularly prone to oxidative damage due to its base composition. Although all DNA bases are susceptible to damage, guanine is most prone to oxidative modification. 8-Hydroxyguanine (8-OH-Gua), and its 2′-deoxynucleoside equivalent, 8-hydroxy-2′-deoxyguanosine (8-OHdG), are the most common byproducts. 8-OH-dG is excreted during the repair of damaged DNA *in vivo*, by exonucleases considering it as a marker of oxidant-induced DNA damage [13,14]. This phenomenon has notable biological consequences, since repair processes directed to non-coding regions are slower and less efficient [9,11,15].

Recent studies have shown that the lung tissue from COPD patients displays oxidative DNA damage [14,16]. The expression of 8-OHdG is significantly increased in the peripheral lung of smokers (with and without COPD) compared with non-smokers while the number of DNA damage and repair sites was increased in smokers compared with non-smokers and patients with COPD, implying the existence of a DNA damage/repair imbalance in COPD [14,16]. Others, using immunohistochemistry, showed that following cigarette smoke exposure, there were increased signals of 8-OHdG in the bronchiolar epithelial cells and the alveolar epithelial cells, particularly type II cells, in animal models [17,18]. Similarly, previous studies of our group have shown that 8-OHdG levels were statistically significantly increased in COPD patients compared to non-COPD smokers and non-smokers and especially in a COPD subgroup with microsatellite instability (MSI) [7].

Other studies mainly focused on determining whether the oxidative DNA damage was randomly distributed or localized in specific sequences in either the nuclear or mitochondrial genomes. It has been shown that oxidative DNA damage in COPD lungs was prominent in the hypoxic response element (HRE) of the Vascular Endothelial Growth Factor (VEGF) promoter, which could contribute to transcriptional deregulation and cell fate decisions in COPD [19].

The oxidative damage in DNA by cigarette smoke is either caused directly, as already discussed, or through the generation of reactive oxygen species (ROS) [20]. ROS oxidatively damage nucleic acids, requiring multiple repair mechanisms, such as base excision pathway components 8-oxoguanine-DNA glycosylase (OGG1), endonuclease III homologue 1 (NTH1), and single-strand-selective monofunctional uracil-DNA glycosylase 1 (SMUG1), as well as the nucleic acid-binding protein, Y-box binding protein 1 (YB1). Studies have shown that ROS-dependent, cigarette smoke-induced nucleic-acid oxidation in alveolar fibroblasts, is involved in the pathogenesis of emphysema [21].

In addition, DNA methylation is shown to regulate the expression of pro-inflammatory genes during the development of COPD. DNA methylation of the promoters of pro-inflammatory genes has been observed both in airway epithelial cells and alveolar macrophages. The DNA methylation status in COPD shows changes similar to that observed in cancer tissues, with hypermethylation of specific promoters and loss of global DNA methylation mainly due to a demethylation in repetitive elements [22–25].

Finally, since aging is characterized by telomeres shortening, the relationship between telomere length and COPD is under investigation. The observation that telomere length in circulating leukocytes of COPD patients are shorter than those of controls, is likely due to the increased systemic oxidative stress, in line with what has been observed in other chronic inflammatory disorders associated to telomere shortening [24,26–29].

## 3. DNA Repair Mechanisms

Direct repair, base excision repair, nucleotide excision repair, double-strand break repair (DSB), and cross-link repair are defined in [30]. The maintenance of chromosomal integrity is dependent on the efficient repair of DNA DSBs [31–33], since those caused by oxidative stress appear to contribute to the pathogenesis of COPD by inducing apoptosis, cell senescence and pro-inflammatory responses [34].

The inefficiency of DNA repair is a common finding in COPD. This could be due to heritable genetic polymorphisms that influence the efficiency of DNA repair mechanisms in both the bronchial epithelium and connective tissue [35,36]. Numerous studies have shown that cigarette smoke causes DNA damage and impaired DSB repair, which indeed is aggravated in patients with COPD/emphysema [14,18,19,21,34,37]. Combined COPD genetic studies have identified XRCC5 as an additional COPD susceptibility gene on chromosome 2q. *XRCC5* is an ATP-dependent DNA helicase involved in DNA double-strand break (DSB) repair and immunoglobulin Variable, Diverse, and Joining [V(D)J] rearrangement, also known as Ku80 or Ku86. This has been implicated in the development of COPD and specifically in early-onset COPD, and also in animal models [38–42].

Interestingly, women have a 10%–15% lower capacity for repairing tobacco induced DNA damage than men [43]. There is also evidence that the DNA repair mechanisms may play an important role in the epigenetic regulation of cell function by mediating DNA demethylation [44].

According to recent evidence, acetylation of histone H3 on lysine 56 AcH3K56 has a critical role in DNA damage and repair [45,46]. Histone deacetylase (HDACs) 2 posttranslational modifications play an important role in the regulation of inflammation, histone/DNA epigenetic modifications, DNA damage response and cellular senescence [47]. Sirtuins also play a crucial role in deacetylating H3K56 in mammalian cells [46,48], suggesting that in diseases such as COPD, where there is a decrease in HDAC2 [49] and SIRT1 (NAD+-dependent protein/histone deacetylase) [50–52] expression, the decreased protection against DNA breakage and repair can be explained together with the increase of somatic mutations. Specifically, the anti-aging SIRT1, that is reduced in lungs of COPD patients, seems to protect against emphysema through FOXO3 (Forkhead box O3)-mediated reduction of cellular senescence, independently of inflammation [53].

Finally, given the important role of mitochondria in the DNA damage and the antioxidant capacity of the human organism, we evaluated the inner mitochondrial membrane proteins (PHB1 and PHB2) in COPD patients [54]. Prohibitins (PHB1 and PHB2) interact with the Nicotinamide adenine dinucleotide hydrogen (NADH) dehydrogenase protein complex, which is essential for oxidoreductase activity within cells. Smoking, due to increased ROS production, damages the mitochondrial respiratory machinery [55–57]. We demonstrated that PHB1 mRNA and protein levels decreased at a linear rate from non-smokers to non-COPD smokers to COPD patients, reflecting a reduced respiratory mitochondrial function, resulting in decreased anti-oxidant capacity, especially in the mitochondria of COPD patients [54].

## 4. DNA Damage—Somatic Mutations in the Microsatellite DNA

Several cellular responses are activated upon DNA damage that enable the cell to either eliminate or reverse the damage or to activate programmed cell death, presumably to eliminate cells with potentially catastrophic mutations. These somatic mutations are usually associated with oncogenes and inflammatory signaling pathways [7,30,58,59].

The oxidative DNA damage in the lung generally affects the base composition of the repeated sequences (microsatellite DNA) especially when coupled with DNA mismatch repair system (MMR) deficiency [11,15].

The microsatellite repeats are identical in the different cells of each individual, and unique. Their characteristics are that they are found scattered throughout the genome, are usually located outside or within coding sequences and are faithfully replicated in healthy cells ensuring that coding sequences remain within the appropriate reading frame [5,11,18]. Frame shift mutations in downstream genes are induced by insertion or deletion of one or more repeat units. They have been used as polymorphic markers used for genome mapping in many organisms, including humans [5,11,18]. The MMR system is important for the maintenance of microsatellite stability, as it is designed to correct single-base mismatches and small insertion/deletion loops that may occur during DNA replication [60]. The study of microsatellite DNA instability (MSI) in lung tissue samples offers an index of acquired mutations and is achieved with the use of microsatellite markers targeting specific chromosomal loci near or in genes that could be implicated in the pathogenesis of a disease [5,11,18].

## 5. Microsatellite DNA Instability in COPD

The induction of MSI by oxidants in COPD has significant biological relevance, given the association of MSI with chronic inflammation, where oxidant production would be enhanced [7].

Ever since the Microsatellite DNA Instability (MI) was established in benign lung diseases, such as COPD and asthma [58,61,62], comparative studies between COPD and asthma at the microsatellite DNA level, revealed that COPD patients had a higher frequency of somatic mutations when compared with asthmatics [63]. Those studies revealed genetic instability in COPD specific microsatellite sites adjacent to genes related to COPD pathogenesis (e.g., Surfactant A, Perforin, cluster of differentiation (CD8), Tumor necrosis factor (TNF)). What was important was to assess the relationship between COPD exacerbations and genetic instability, since the “frequent COPD exacerbator” exhibited the highest rates of genetic defects, implying that persistent inflammation and increased oxidative burden on cells during exacerbations, could lead to greater oxidative DNA damage when compared with stable COPD [64].

What was next to be explored was the identification of the susceptible sputum cell subpopulation to acquired somatic mutations. Studies from our group were focused on the lung epithelial barrier cells (LEBCs), which constitute the outer cellular layer of the bronchial tree and are exposed to numerous host and environmental insults [65]. The air–lung barrier system, the first line of defense according to numerous studies, is constantly affected by the burden of inhaled oxidants from cigarette smoking and the increased amount of reactive oxygen species generated by various inflammatory cells of the airways in COPD [1,2]. Using immuno-magnetic beads for sputum cell separation followed by flow cytometry and microsatellite DNA analysis, Samara *et al.* verified that somatic DNA alterations were exclusively exhibited in the lung epithelial barrier cells of COPD patients [65].

## 6. DNA Damage Due to Oxidative Stress in COPD: A Hypothesis

Based on current studies, we have already proposed a new hypothesis on the pathogenesis of COPD including the oxidative DNA damage of the lung epithelial barrier cells (LEBCs) in COPD [8].

In the initiation of COPD, cigarette smoking affects the air-lung barrier system and, especially, the epithelium via repeated oxidative stress leading to oxidative DNA damage [2,10,15]. The “danger hypothesis” of Matzinger, further explains how cigarette smoke and oxidative stress triggers the innate immune response. According to her theory, it is not only the presence of an external antigen (e.g., pathogens, toxins, smoke) that alerts the immune system to respond but also self-antigens (endogenous intracellular molecules or danger-associated molecular patterns from stressed or dying cells) [66]. These molecular patterns or self-antigens are being recognized by Toll-like receptors (TLRs), the sensors of the innate immune system [67].

Cigarette smoke contains more than 2000 xenobiotic compounds and 10^14^ free radicals that injure lung epithelial cells and cause the breakdown of connective tissue in smokers [68,69]. Epithelial cell injury products can act as ligands for TLRs, inducing epithelial cells to produce inflammation mediators. These mediators activate alveolar macrophages and neutrophils [70,71], which in turn secrete proteolytic enzymes and, together with reactive oxygen species, further damage lung tissue [72]. The polarised cells travel then to the draining lymph nodes and present danger signals to the naive T-lymphocytes, inducing a proliferation of CD8+ cytotoxic T-lymphocytes. The CD8+ T-cells, in turn, migrate to the sites of the initial insult, releasing perforin and granzymes and attack the altered LEBCs activating cell death cascades [4,8,73,74].

Oxidative stress, cell death and tissue trauma could release self-antigens, damage mitochondria, modify proteins and release truncated DNA from apoptotic cells [75–77]. These products can be recognized by the adaptive immune system as foreign antigens, triggering an immune reaction. Similarly, in smokers, such antigens have been shown to be released as a result of necrosis and apoptosis of epithelial and endothelial cells and extracellular-matrix injury [76–81]. On the other hand, Toll-like Receptors (TLRs) link innate and adaptive immunity, enhancing the pathogenic potential of those antigens [82–85].

Besides, the damaged DNA of LEBCs due to oxidative stress, leads to increased expression of 8-OHdG, further generation of ROS, decreased DNA auto-repair ability and finally somatic mutations [7,14,15,17,18,20,30,36,65]. Evidently, after smoking cessation, oxidative stress and somatic mutations persist in a number of COPD patients, perpetuating pulmonary inflammation.

The DNA damage response (DDR), the cellular response that allows DNA damage detection, signaling and repair is stimulated at any place and any time, where and when DNA lesions occur [10,86,87]. Oxidative DNA damage, DSBs and HDAC2 posttranslational modifications have recently been shown to induce DDR, which in turn causes chronic inflammation in COPD as a result of profound cellular changes to the senescence-associated secretory phenotype (SASP). The SASP can disrupt normal tissue structures and function [34,47,88]. Senescence of alveolar epithelial cells limits cell proliferation causing increased production of pro-inflammatory cytokines, hence promoting inflammation. When alveolar epithelial cells reach the senescent stage, the lungs suffer both from impaired alveolar regeneration and exaggerated alveolar inflammation, thereby contributing to COPD pathogenesis [41,89,90].

As already discussed, the cells of COPD patients, in response to oxidative stress, present reduced HDAC2 activity which has been linked to increased acetylation of the antioxidant transcription factor Nuclear respiratory factor 2 (Nrf 2), impairing Nrf 2 activity, and to the upregulation of antioxidant enzymes [91]. Moreover, the impairment of Nrf2-mediated antioxidant defenses, along with epigenetic changes induced by ROS (histone modifications) and mitochondrial dysfunction, seems to prolong the oxidative stress in COPD [49,92,93].

## 7. Conclusion

In conclusion, oxidative DNA damage contributes to the molecular pathogenesis of COPD. Increased 8-OHdG expression and acquired somatic mutations in COPD are the result of the oxidative DNA damage and the inefficient DNA repair machinery. Moreover, since the SASP is largely a response to genomic or epigenomic damage, it may be a model for a cellular damage response that can propagate damage signals both within and among tissues. Since COPD is thought to be a degenerative disease of aging, it may be fueled by such damage signals. The varying efficiency of DNA repair across the genome may be viewed as analogous to inter-individual variation in repair efficiency, a potential determinant of disease susceptibility.

## Supplementary Information


