# Supplementary material for: DNA Damage Due to Oxidative Stress in Chronic Obstructive Pulmonary Disease (COPD)
Source: Int J Mol Sci. 2012 Dec 10;13(12):16853–64. doi: 10.3390/ijms131216853 (PMC3546726; doi:10.3390/ijms131216853)
Supplement: Supplementary file 1 [file ijms-13-16853-s001.pdf]

Supplementary Information

Figure S1. Oxidative DNA damage in COPD can be primarily induced by cigarette smoking and the recruited immune and inflammatory cells. The consequences of oxidative DNA damage could comprise 8-OHdG excretion, somatic mutations (such as Microsatellite DNA instability), mitochondrial dysfunction, histone deacetylation, DNA methylation, telomere shortening. These events, thus, trigger immune responses, cell death, and chronic inflammation leading to COPD.

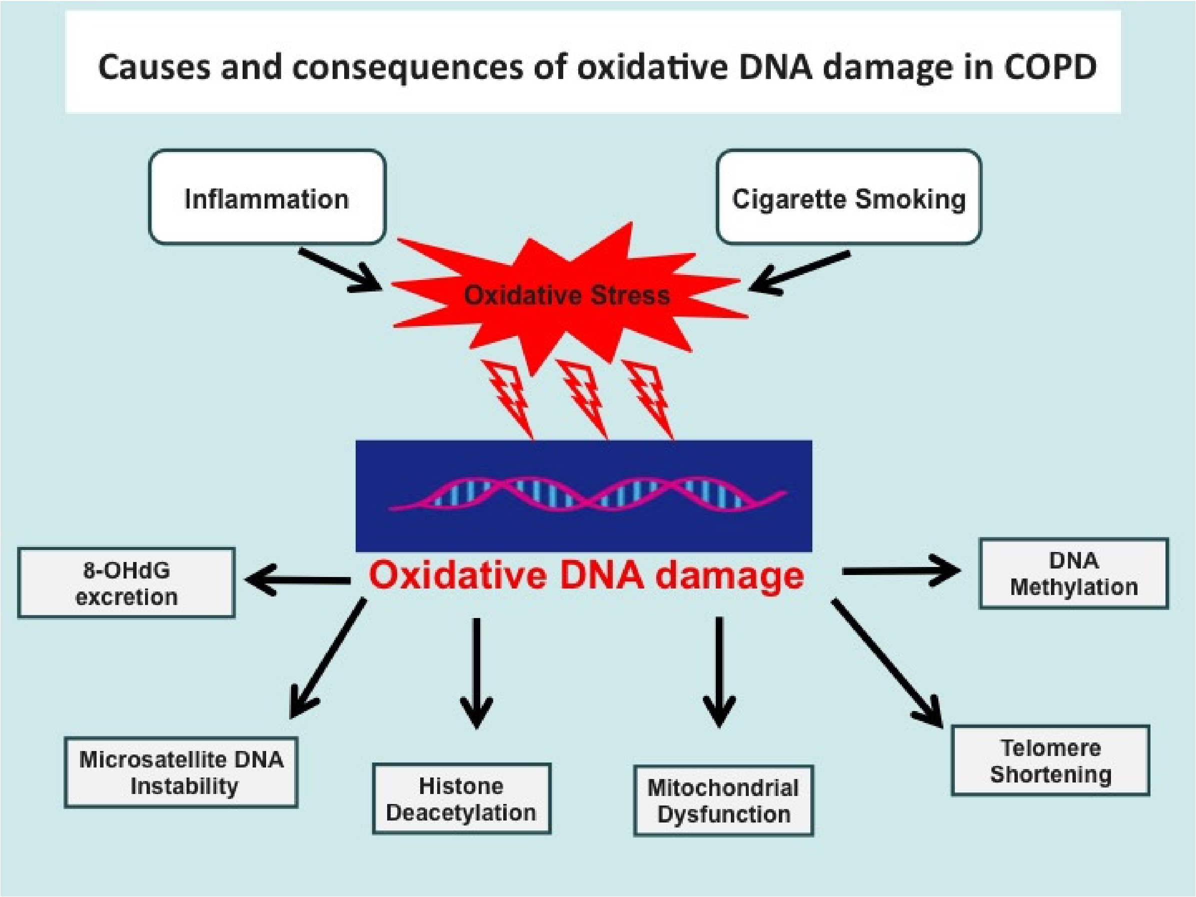

© 2012 by the authors; licensee MDPI, Basel, Switzerland. This article is an open access article distributed under the terms and conditions of the Creative Commons Attribution license (http://creativecommons.org/licenses/by/3.0/).
